# Cognitive Behavioral Therapy for Reproductive Concerns and Psychological Status in Reproductive‐aged Breast Cancer Survivors: A Pretest‐Posttest Intervention Study

**DOI:** 10.1002/brb3.71065

**Published:** 2025-11-14

**Authors:** Maedeh Rezaei, Hamed Milani, Mohsen Kheiri, Hadis Tolomehr, Zohreh Shahhosseini, Marzieh Azizi

**Affiliations:** ^1^ Department of Midwifery Go.C., Islamic Azad University Gorgan Iran; ^2^ School of Medicine Mazandaran University of Medical Sciences Sari Iran; ^3^ School of Pharmacy Mazandaran University of Medical Sciences Sari Iran; ^4^ School of Paramedical Sciences Mazandaran University of Medical Sciences Sari Iran; ^5^ Department of Midwifery, Sexual and Reproductive Health Research Center Mazandaran University of Medical Sciences Sari Iran

**Keywords:** BC, CBT, fertility, psychological status, reproductive‐aged, survivors

## Abstract

**Introduction:**

Reproductive‐aged breast cancer (BC) survivors face significant distress related to fertility concerns and psychological health, yet interventions specifically targeting these issues are lacking.

**Objective:**

This study aimed to evaluate the effectiveness of a cognitive‐behavioral therapy (CBT) intervention on fertility concerns and psychological status in this population.

**Design:**

A quasi‐experimental pretest‐posttest study.

**Methods:**

This study was conducted with 30 premenopausal BC survivors who desired future pregnancy. Participants were recruited via convenience sampling from a hospital and a clinic in Sari, Northern Iran. They underwent a six‐week, group‐based, face‐to‐face CBT program delivered by a reproductive health specialist. Fertility concerns were measured using the reproductive concerns after cancer (RCAC) scale, and psychological status was assessed with the depression, anxiety, and stress Scales (DASS‐21) at baseline and immediately post‐intervention. Data were analyzed using paired t‐tests and the Bonferroni correction using in SPSS 24.

**Results:**

The CBT intervention resulted in statistically significant reductions in scores across five of the six RCAC subscales: concerns about fertility potential, partner disclosure, child's health, personal health, and becoming pregnant (*p* < 0.001). Concerns regarding acceptance of possible infertility did not show a significant change (*p* = 0.628). Furthermore, participants demonstrated significant decreases in symptoms of depression (*p* < 0.001), anxiety (*p* < 0.001), and stress (*p* = 0.031) on the DASS‐21.

**Conclusion:**

A structured CBT intervention is an effective short‐term strategy for alleviating specific fertility‐related concerns and improving the psychological well‐being of premenopausal BC survivors. The findings support the integration of psychological support, particularly CBT, into survivorship care programs to address the complex reproductive and mental health challenges faced by this group. Further research with a control group and long‐term follow‐up is recommended to confirm these findings.

## Graphical abstract

This pretest‐posttest intervention study evaluated a structured, six‐week CBT program for premenopausal BC survivors desiring pregnancy. The intervention led to significant, large‐magnitude improvements, effectively reducing distress across five key areas of fertility concern including fertility potential, partner disclosure, and pregnancy—and significantly alleviating symptoms of depression, anxiety, and stress. The findings provide strong preliminary evidence that targeted CBT is a potent short‐term strategy to enhance psychological well‐being and address the complex reproductive challenges faced by this population, advocating for its integration into standard survivorship care.

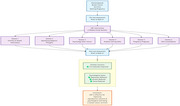

## 1 Introduction

BC is the most prevalent cancer and the second leading cause of cancer‐related mortality among women globally (Azizi et al. 2024). In Iran, BC is the most common type of cancer and the fifth leading cause of death due to malignancy (Jafari et al. 2018). In recent decades, the increasing trend of delaying childbearing to later reproductive years has led to a rise in the number of women seeking pregnancy after the age of 35 (Córdoba et al. 2012; Ives et al. 2012). This issue also applies to reproductive‐aged BC survivors who have not completed their desired family size before their diagnosis (Ives et al. 2007). According to a study in the United States, 25% of women with BC are premenopausal, of whom 7% are under 40 at the time of diagnosis (Calhoun and Hansen 2006). The results of a study showed that delayed childbearing in women with BC does not alter cancer‐related outcomes or pregnancy‐related complications. However, many affected women prefer to postpone pregnancy until completing their treatment (Ives et al. 2012).

The literature review revealed that BC survivors face multiple challenges, including psychological distress, sexual dysfunction, body image concerns, marital relationship difficulties, and unmet informational needs (Ives et al. 2007). In other words, BC is a profoundly challenging experience accompanied by physical, psychological, and social consequences (Henry et al. 2012). Factors such as high stress levels, drug‐induced toxicity, and the adverse effects of chemotherapy and radiotherapy have long‐term detrimental impacts on female fertility and sexual function. Consequently, a substantial proportion of these patients experience infertility after cancer treatment (Ghaemi et al. 2019; Rodriguez‐Wallberg et al. 2023). Thus, fertility preservation has become an increasingly critical component of therapeutic strategies for BC patients (Córdoba et al. 2012; Ives et al. 2012). However, for women with BC who still desire childbearing, fertility preservation may present a significant clinical challenge (Ruggeri et al. 2019).

BC survivors who became mothers after completing treatment reported that motherhood helped restore their sense of well‐being and normalcy. However, they simultaneously experienced profound anxieties, including concerns about their child's health, heightened fears of disease recurrence post‐pregnancy, worries about their long‐term survival to raise their child properly, and doubts about their capacity to fulfill maternal responsibilities adequately (Harirchi et al. 2011).

Due to the profound impact of fertility concerns on multiple aspects of life for reproductive‐aged women, research into interventions that enhance the psychological well‐being of BC survivors is critically essential. Psychological treatments such as CBT are among the therapeutic interventions whose effectiveness has been proven in various fields (Azizi et al. 2024). CBT is a short‐term treatment that focuses on individuals' thoughts about their situation and the emotions they experience, which are influenced by their way of thinking. Additionally, this intervention helps individuals recognize how destructive thoughts, feelings, and attitudes affect their behavior (Faraji et al. 2015; Karamoozian 2014).

While CBT and other psychosocial interventions have been shown to improve the general psychological well‐being of BC patients (Elyasi et al. 2021; Sun et al. 2019), and recent studies have begun to explore specific interventions for reproductive concerns such as couple‐based counseling (Barjasteh et al. 2022) and fertility‐focused decision‐making aids, few studies have explicitly evaluated the efficacy of a structured, group‐based CBT protocol specifically designed to address the multifaceted nature of fertility concerns in reproductive‐aged BC survivors. The present study, therefore, aimed to evaluate the effectiveness of such a targeted CBT intervention on both fertility concerns and broader psychological status in this patient group.

## 2 Materials and Methods

### 2.1 Study design

A quasi‐experimental pre‐test, post‐test design was conducted to evaluate the fertility concerns and psychological status of BC survivors before and after CBT.

### 2.2 Setting and Participants

This study was conducted on 30 reproductive‐aged BC survivors, selected from a referral hospital and specialty and superspecialty clinics in Sari, Mazandaran, Iran, using a convenience sampling method.

### 2.3 Inclusion and Exclusion Criteria

The inclusion criteria were: reproductive‐aged women with a desire to become pregnant after completion of their treatment and surviving based on self‐report, permission from their oncologist to attempt pregnancy, literate, no current diagnosis of a psychiatric disorder, no addiction to alcohol or drugs, and no consumption of psychotropic medications. In contrast, unwillingness to continue participation in the study or absence from two or more intervention sessions were considered exclusion criteria in this study.

### 2.4 Sample Size

The sample size was calculated a priori for a paired‐sample t‐test using GPower software (version 3.1). The calculation was based on the primary outcome of fertility concerns (total RCAC score). Input parameters were derived from a previous intervention study with a comparable population and outcome (Elyasi et al. 2021), which yielded an effect size (Cohen's d) of approximately 0.80. With an alpha (α) of 0.05 and a desired power (1‐β) of 0.90, the minimum required sample size was 18 participants. To account for a potential 20% attrition rate, we aimed to recruit a final sample of 22 participants. However, during the recruitment period, we were able to enroll 30 eligible and consenting participants, which exceeded our initial target and provided enhanced power for the analysis.″*

### 2.5 Instruments

In this study, the data collection instruments consisted of a demographic and clinical characteristics questionnaire, the RCAC, and the DASS‐21.

### 2.6 Demographic and Clinical Characteristics Questionnaire

This tool included items such as participants' age, age at marriage, age at the time of cancer diagnosis, duration of illness, participant's educational level, spouse's education level, occupation, spouse's occupation, income adequacy, family history of BC, breast‐conserving surgery, type of surgery, adjuvant therapy type, number of pregnancies before and after BC diagnosis, number of children before and after BC diagnosis, and history of abortion before and after BC diagnosis.

### 2.7 RCAC Scale

The RCAC scale was developed by Gorman et al. in 2014 to identify a range of fertility and parenting concerns, as well as unmet needs, experienced by reproductive‐aged cancer survivors (Gorman et al. 2014). This scale consists of 18 items rated on a 5‐point Likert scale. The instrument comprises six subscales: “concerns about fertility potential,” “partner disclosure of fertility status,” “child's health,” “personal health”, ”acceptance of possible infertility,“ and “becoming pregnant,” with each subscale containing three items. The tool was designed based on a qualitative analysis of focus group discussions and individual interviews with young cancer survivors aged 18 to 34. According to the study's results, the reliability of this tool, as measured by internal consistency for the total scale score, was confirmed with a Cronbach's alpha coefficient of 0.82. For each of the three‐item subscales, coefficients ranged from 0.78 to 0.88. It was also demonstrated that the instrument possesses acceptable convergent and divergent validity (Alpha = 0.82) (Gorman et al. 2019). In addition, the validity and reliability of this instrument in the Iranian population were examined, and its reliability was confirmed with a Cronbach's alpha of 0.87 (Barjasteh et al. 2022). In the present study, the internal consistency for the RCAC total scale was excellent (Cronbach's α = 0.88).

### 2.8 DASS‐21

DASS‐21 comprises a set of three self‐report scales designed to measure the emotional states of depression, anxiety, and stress (Bottesi et al. 2015). This questionnaire is the short form of the 42‐item DASS, developed by Lovibond et al. in 1995. Each subscale contains seven items. Scoring for each item is based on a 4‐point Likert scale: 0 (“Did not apply to me at all”) to 3 (“Applied to me very much, or most of the time”) (Basha and Kaya 2016). The minimum score for each subscale is 0, and the maximum is 21. A higher score on the three subscales indicates a worse psychological status. The reliability of the DASS‐21 was demonstrated by excellent Cronbach's alpha values of 0.81, 0.89, and 0.78 for the subscales of depression, anxiety, and stress, respectively (Coker et al. 2018). In an Iranian population, the validity and reliability of this tool were confirmed with Cronbach's alpha coefficients of 0.79 for the anxiety subscale, 0.91 for stress, and 0.93 for depression. Furthermore, the internal consistency coefficient for all dimensions was calculated to be between 0.75 and 0.86, indicating the acceptable reliability of this scale (Kakemam et al. 2022). In the present study, the internal consistency for the DASS‐21 was excellent (Cronbach's α = 0.91). The subscales also demonstrated good reliability: Depression (α = 0.85), anxiety (α = 0.82), and stress (α = 0.79).

### 2.9 Intervention

Face‐to‐face CBT intervention sessions were conducted by a reproductive health specialist (M.A.), who holds a Master of Science in Counseling with a focus on Midwifery. The therapist had received formal graduate‐level training in CBT and completed a specialized workshop on CBT in psychosocial oncology to ensure competence. To guarantee intervention fidelity, a detailed session manual was used, and the principal investigator monitored adherence through periodic reviews of session audio recordings. The sessions were held for two groups of 15 participants, each consisting of six weekly sessions lasting 70 min. Participant attendance was recorded at each session; no participant missed more than one session (96% attendance rate), and over 90% consistently completed the weekly homework assignments to monitor engagement.

In summary, each session included a description of the CBT, a review of the previous session's content, a question‐and‐answer period, and homework assignments for the following week. At the beginning of the study, during the pre‐test phase, the participants completed the mentioned questionnaires. Immediately after the intervention, questionnaires were also completed by these groups. The content of the CBT sessions is shown in Table 1. In this study, we did not assign a control group.

**TABLE 1:** Overview of CBT sessions for BC survivors.

Session	Session guide	Session objectives	Session contents	Intervention techniques used
Session 1	‐ Introductions/welcome ‐ BC discussion: symptoms/fertility impacts ‐ Q and A ‐ Homework assignment ‐ Questionnaire completion	‐ Establish participant rapport. ‐ Provide required information regarding BC and disease symptoms. ‐ Explain the impact of cancer on fertility.	‐ BC information: symptoms, risk factors, diagnosis, treatment. ‐ Introduction to CBT concepts. ‐ Identifying ANTs and the relation between thoughts, emotion, and behavior.	‐ ANT identification techniques and their forms. ‐ The technique of distinguishing between thoughts and feelings.
Session 2	‐ Attendance ‐ Homework review ‐ Common survivor concerns ‐ Q and A ‐ Next assignment	‐ Address common survivor concerns. ‐ Pregnancy after cancer treatment. ‐Common pregnancy complaints and their management. ‐ Teaching the principles of CBT, NATs, and how to identify them.	‐ Common post‐cancer fertility concerns. ‐ Pregnancy rates after BC. ‐ Further familiarize with the concept of ANT and the connection between thoughts, feelings, and behavior.	‐ Teaching the technique of distinguishing thoughts from feelings. ‐ Teaching critical examination technique. ‐ Teaching problem‐solving training.
Session 3	‐ Attendance /homework review ‐ Psychological challenges ‐ Q and A ‐ Next assignment	‐ Importance of psychological health in pregnancy. ‐ The common psychological issues among pregnant BC survivors. ‐ Challenging NATs and/or cognitive distortions in women regarding the experience of pregnancy and childbearing, and replacing them with accurate thoughts.	‐ Common psychological issues in BC survivors. ‐ Challenging ANTs in BC survivors regarding the experience of pregnancy and childbearing.	‐ Catastrophizing prevention along with problem‐solving. ‐ Problem distancing education. ‐ Survivor pregnancy experience and answer participants' questions. ‐ Showing the short videos.
Session 4	‐ Attendance ‐ Previous session review ‐ Psychological impacts ‐ Q and A ‐ Next assignment	‐ Mental health effects on pregnancy. ‐ Cognitive distortion education.	‐ Anxiety/depression effects on maternal and fetal health during pregnancy. ‐ CBT‐based cognitive distortions.	‐ Small group discussions (5 people). ‐ Cognitive distortion identification and its importance in reinforcing NATs. ‐ Showing the short videos.
Session 5	‐ Attendance ‐ Session review ‐ Pregnancy's impact on cancer prognosis impact ‐ Q and A (10 min) ‐ Next assignment	‐ Review previous content. ‐ Pregnancy‐cancer prognosis evidence ‐ CBT skill application.	‐Review of the material covered in the past four sessions. ‐Information regarding the impact of pregnancy on cancer prognosis based on the latest available scientific evidence. ‐ Fertility challenge case study and engage in a group discussion to provide solutions and resolve the challenge based on the CBT they have learned so far.	‐ Small group problem‐solving and intra‐group discussion. ‐ Guided Q and A
Session 6	‐ Attendance ‐ Program review/Q and A ‐ Questionnaire completion	‐ Curriculum reinforcement ‐ Skill consolidation ‐ Participant empowerment	‐Review of CBT through your active participation in presenting materials and explaining the use cases of these strategies in different situations. ‐Review of the entire sixth session and session summary.	‐ Q and A session ‐ Survivor birth experience sharing

Abbreviaitons: Q and A: Question & answer; ANTs: automatic negative thoughts.

### 2.10 Data Analysis

Data were analyzed using SPSS version 24 (IBM Corp., Armonk, NY, USA). Descriptive statistics are presented as mean ± standard deviation (SD) for continuous variables and as frequency (percentage) for categorical variables. The normality of the distribution of scale scores was confirmed using the Shapiro‐Wilk test. Given that all outcome variables were normally distributed, pre‐ and post‐intervention comparisons were conducted using paired samples t‐tests. Effect sizes were calculated using Cohen's *d*, interpreted as small (0.2), medium (0.5), and large (0.8). Due to the multiple comparisons across the six RCAC and three DASS‐21 subscales, a Bonferroni correction was applied. The significance level of *p* < 0.05 was retained for descriptive purposes and to inform effect sizes.

### 2.11 Ethical Considerations

Following approval from the Mazandaran University of Medical Sciences Council and Ethics Committee (ethical code: IR.MAZUMS.REC.1404.349), this study was conducted and reported in accordance with the TREND (Transparent Reporting of Evaluations with Nonrandomized Designs) guidelines. Participants were briefed on the study's objectives, intervention structure, and session details. They were assured of the confidentiality and anonymity of their data and that their participation was voluntary. Informed written consent was subsequently obtained from all participants in line with the Declaration of Helsinki.

## 3 Results

### 3.1 Demographic and Clinical Characteristics of the Participants

The demographic and clinical characteristics of the participants are shown in Table 2. The study included 30 BC survivors with a mean age of 35.10 ± 4.02 years. The majority of participants had a high school education or less (70.0%), were houseworkers (76.7%), and reported moderate income adequacy (53.3%). Similarly, most of their spouses had a high school education or less (60.0%) and were self‐employed (66.6%).

**TABLE 2:** The demographic and clinical characteristics of the participants (*n* = 30).

Variable	mean± SD or frequency (%)
Participants' age	35.10 ± 4.02
Age at marriage	25.67± 3.17
Age at time of cancer diagnosis	29.57 ± 3.41
Duration of illness (years)	4.00 ± 1.53
Educational level High school or lower Academic	21 (70.0) 9 (30.0)
Spouse's education level High school or lower Academic	18 (60.0) 12 (40.0)
Occupation Houseworker Employed	23 (76.7) 7 (23.3)
Spouse's occupation Self‐employed Employed Retired	20 (66.6) 8 (26.7) 2 (6.7)
Income adequacy Low Moderate High	7 (23.3) 16 (53.3) 6 (20.0)
Family history of breast cancer Yes	12 (40.0)
Breast‐conserving surgery Yes	13 (43.3)
Type of surgery Total mastectomy Lumpectomy	5 (16.7) 8 (26.7)
Adjuvant therapy type Chemotherapy Radiotherapy Chemotherapy + Radiotherapy Hormone Therapy	8 (26.7) 1 (3.3) 4 (13.3) 15 (50.5)
Number of pregnancies before diagnosis 0 1 2	15 (50.0) 11 (36.7) 4 (13.3)
Number of children before diagnosis 0 1 2	18 (60.0) 11 (36.7) 1 (3.3)
History of abortion before diagnosis Yes	5 (16.6)
Number of abortions before diagnosis 1 2	4 (13.3) 1 (3.3)
Number of pregnancies after diagnosis and treatment 0 1 2	19 (63.3) 9 (30.0) 2 (6.7)
Number of Children After Diagnosis and treatment 0 1	21 (70.0) 9 (30.0)
History of abortion after diagnosis and treatment Yes	3 (10.0)
Number of abortions after diagnosis and treatment 1 2	2 (6.7) 1 (3.3)

Abbreviations: SD, standard deviation. Data are presented as mean ± SD for continuous variables and as frequency (percentage) for categorical variables.

Participants were diagnosed with BC at a mean age of 29.57 ± 3.41 years and had a mean illness duration of 4.00 ± 1.53 years at the time of the study. A family history of BC was reported by 40.0% of the participants. In terms of treatment, 43.3% underwent breast‐conserving surgery, with lumpectomy (26.7%) being more common than total mastectomy (16.7%). Hormone therapy was the most prevalent adjuvant treatment (50.5%), followed by chemotherapy alone (26.7%).

Regarding the obstetrics history, before their diagnosis, 60.0% had no children. A history of abortion before BC diagnosis was reported by 16.6% of participants. Following their cancer treatments and surviving, 30% had one, 6.3% had experienced two pregnancies, and 30.0% had no children. Among those who did conceive post‐diagnosis, 10.0% reported a history of abortion.

The results of the paired t‐tests, as detailed in Table 3, demonstrated that the CBT intervention led to statistically significant reductions in fertility concern scores across five of the six subscales. Significant decreases were observed in concerns about fertility potential (*t* = 5.979, *p* < 0.001, *d* = 1.09), partner disclosure of fertility status (*t* = 9.060, *p* < 0.001, *d* = 1.65), child's health (*t* = 7.573, *p* < 0.001, *d* = 1.38), personal health (*t* = 6.113, *p* < 0.001, *d* = 1.12), and becoming pregnant (*t* = 12.022, *p* < 0.001, d = 2.19). In contrast, the intervention did not yield a significant reduction in concerns regarding the acceptance of possible infertility (*t* = 0.490, *p* = 0.628, *d* = 0.09).

**TABLE 3:** Comparison of fertility concern scores (RCAC) in BC survivors before and after the intervention.

Subscale	Mean ± SD (before)	Mean ± SD (after)	Mean difference (before—after)	95% confidence interval of the difference	t	*p*‐value (uncorrected)	*p*‐value (Bonferroni‐corrected)*	Effect size (Cohen's d)
Fertility potential	11.73 ± 1.84	8.83 ± 1.45	2.90	1.91 to 3.89	5.979	< 0.001	< 0.001	1.09
Partner disclosure of fertility status	10.67 ± 2.80	6.27 ± 2.10	4.40	3.41 to 5.39	9.060	< 0.001	< 0.001	1.65
Child's health	11.90 ± 1.54	8.63 ± 2.30	3.27	2.39 to 4.15	7.573	< 0.001	< 0.001	1.38
Personal health	10.93 ± 1.91	7.70 ± 2.00	3.23	2.16 to 4.30	6.113	< 0.001	< 0.001	1.12
Acceptance of possible infertility	8.87 ± 2.66	8.60 ± 1.45	0.27	−0.85 to 1.39	0.490	0.628	1.000	0.09
Becoming pregnant	11.60 ± 1.43	8.73 ± 1.36	2.87	2.38 to 3.36	12.022	< 0.001	< 0.001	2.19

*Notes: The significance level was adjusted for multiple comparisons (9 tests) using the Bonferroni method.

As shown in Table 4, the intervention also resulted in significant improvements in psychological status. Participants exhibited statistically significant decreases in symptoms of depression (*t* = 4.962, *p* < 0.001, *d* = 0.91) and anxiety (*t* = 4.120, *p* < 0.001, *d* = 0.75). The stress reduction was statistically significant before correction (*t* = 2.268, *p* = 0.031, *d* = 0.41); however, this result did not survive the Bonferroni correction (corrected *p* = 0.279).

**TABLE 4:** Comparison of depression, anxiety, and stress scores in BC survivors before and after the intervention.

Subscale	Mean ± SD (before)	Mean ± SD (after)	Mean difference (before—after)	95% confidence interval of the difference	t	*p*‐value (uncorrected)	*p*‐value (Bonferroni‐corrected)*	Effect size (Cohen's d)
Depression	9.80 ± 4.51	6.80 ± 1.92	3.00	1.76 to 4.24	4.962	< 0.001	< 0.001	0.91
Anxiety	6.70 ± 2.82	5.10 ± 1.81	1.60	0.81 to 2.39	4.120	< 0.001	0.001	0.75
Stress	13.17 ± 3.13	12.00 ± 1.91	1.17	0.12 to 2.22	2.268	0.031	0.279	0.41

BC, breast cancer; SD, standard deviation. *Paired samples t‐test was conducted for each subscale. The *p*‐values were corrected for multiple comparisons using the Bonferroni method (corrected α = 0.0167 for three tests).*

## 4 Discussion

This study aimed to evaluate the effectiveness of a CBT intervention for fertility concerns and psychological status in reproductive‐aged BC survivors. The results demonstrated that the six‐week, group‐based CBT program led to statistically significant and large‐magnitude improvements across most domains of fertility concerns, including worries about fertility potential, partner disclosure, child's health, personal health, and becoming pregnant. In other words, the CBT sessions effectively reduced fertility concerns in BC survivors with childbearing intentions following the intervention.

Our results are consistent with a growing recognition of the need for targeted psychosocial support for cancer‐related infertility distress. For instance, a recent randomized controlled trial by Barjasteh et al. (2022) demonstrated that a couple‐based counseling model significantly reduced fertility concerns in BC survivors, highlighting the importance of addressing this issue. Our study builds upon this emerging literature by providing preliminary evidence for the efficacy of a structured, group‐based CBT framework, which focuses on modifying the cognitive patterns underlying these concerns. While the intervention modalities differ, the positive outcomes from both studies underscore the value of providing specialized psychological interventions to alleviate the reproductive distress experienced by young cancer survivors. The significant reductions in fertility concern scores align with qualitative findings that women's pregnancy‐related anxieties often involve fears of disease recurrence, pregnancy loss, and impact on fetal health (Vanstone et al. 2021). Concerns about fertility may influence treatment decision‐making among young cancer patients. In a recent study, 12% of long‐term BC survivors diagnosed at age 40 or younger reported that fertility considerations impacted their treatment decisions. Results from a web‐based survey of young cancer survivors indicated that fertility concerns influenced their treatment decisions in approximately 30% of cases (Ah 2004). The results of a study that aimed to determine the quality of life (QoL), fertility concerns, and mental health outcomes in young BC survivors showed that these women experienced worse QoL and depressive symptoms compared with both the cancer‐free population and survivors over 50 years old. Concerns about premature menopause, menopausal symptoms, and infertility were more prevalent among younger women and played a significant role in post‐treatment distress (Howard‐Anderson et al. 2012). According to the results of a study exploring differences in fertility‐related concerns between BC survivors and non‐cancer infertile women, these women presented identical concerns to the non‐cancer infertile group and higher than the healthy women about fertility potential. Overall, the uncertainty about post‐cancer fertility can be profoundly disruptive, leaving BC survivors at risk for future emotional distress due to their significant fertility concerns (Bártolo et al. 2020).

The results of this study also indicated that scores for depression and anxiety significantly decreased after the intervention. Additionally, stress levels also showed a statistically significant reduction following the intervention. Strong empirical support for the use of CBT for common psychological problems in physical illnesses is entirely consistent with the provision of modern healthcare and its emphasis on empirically supported treatments. To date, cognitive‐behavioral models and their associated treatment protocols have been developed for a wide range of psychological disorders and chronic medical conditions, including cancer. Many of these approaches have demonstrated effectiveness in clinical research (Pedram et al. 2011). Consistent with the findings of the present study, an RCT study that aimed to determine the efficacy of mindfulness‐based cognitive therapy on psychological symptoms in BC survivors demonstrated a reduction in depression, stress, and anxiety symptoms among the survivors (Nabipoor Gisi et al. 2019). A meta‐analysis investigating the efficacy of CBT on QoL and psychological symptoms in BC patients and survivors, based on the results of 10 included studies, confirmed that CBT is an effective method for improving depression, anxiety, and stress symptoms in women with and surviving BC (Ye et al. 2018). Consistent with the findings of the present study, a quasi‐experimental study aimed to determine the effectiveness of cognitive‐behavioral group therapy (CBGT) on reducing depression, anxiety, and stress in women with BC demonstrated that CBGT significantly reduced depression, anxiety, and stress in women with BC (Khatibian and Shakerian 2014). Furthermore, the results of a RCT which aimed to determine the effectiveness of CBT on depression and anxiety symptoms in Chinese women with BC, investigated three groups CBT, self‐care management, and routine care (CBT and self‐care management groups received nine therapy sessions over 12 weeks) indicated that women in the CBT group showed significantly fewer symptoms of depression and anxiety compared to women in the other two groups (Ren et al. 2019).

This study has several important limitations that must be considered. The most significant limitation of this study is its quasi‐experimental, single‐arm design, which lacks a control group. This design was primarily necessitated by the practical difficulties in recruiting a sufficiently large sample of this specific, hard‐to‐reach population (premenopausal BC survivors desiring pregnancy) for a controlled trial within a single center. Consequently, while the pre‐post improvements are promising and statistically significant, we cannot rule out the influence of confounding variables such as the natural progression of adjustment over time or the therapeutic benefits of group participation. Furthermore, the generalizability of our findings is constrained by several factors related to the sample. First, the sample size, though sufficient for our pre‐post analysis, remains relatively small. Second, using a convenience sample from a single geographic location in Northern Iran means our participants may not be representative of the broader population of BC survivors, particularly those from different cultural or healthcare contexts. In addition, as participants were self‐selected and willing to engage in a psychological intervention, they may represent a subgroup with higher motivation or specific concerns, potentially introducing selection bias. These factors limit the external validity of our results, and future multi‐center studies with larger, randomized samples are needed to confirm the general applicability of our intervention.

Furthermore, the sustainability of the intervention's effects remains unknown. The lack of long‐term follow‐up assessments after the immediate post‐intervention measurement means we cannot determine whether the significant improvements in fertility concerns and psychological distress were maintained over subsequent weeks or months. This is a critical question for clinical application, as the challenges of survivorship and family planning are ongoing.

## 5 Conclusion

The findings of this study provide preliminary evidence for the short‐term efficacy of a structured, group‐based CBT intervention for alleviating specific fertility concerns and improving the psychological well‐being of premenopausal BC survivors. The intervention led to significant, large‐effect‐size reductions in core reproductive worries, including concerns about fertility potential, partner disclosure, and becoming pregnant and concurrently ameliorated symptoms of depression and anxiety. The one domain that did not show improvement, “acceptance of possible infertility,” highlights a potential area for future intervention refinement, suggesting that deeper existential concerns may require complementary therapeutic approaches. However, it is crucial to interpret these results within the context of the study's limitations, notably the single‐arm design and small sample size. Therefore, in conclusion, the findings from this pilot study provide promising preliminary evidence that a structured, group‐based CBT intervention may be an effective strategy for alleviating specific fertility‐related concerns and improving psychological well‐being in this population. The significant pre‐post improvements justify and inform the design of a future multi‐center RCT, which is essential to robustly establish causal efficacy and generalizability.

## Author Contributions

**Marzieh Azizi**: conceptualization, validation, investigation, project administration. **Zohreh Shahhosseini**: conceptualization, validation, investigation, project administration. **Maedeh Rezaei**: writing – original draft, methodology. **Marzieh Azizi**: writing – original draft, methodology. **Hamed Milani**: conceptualization, methodology, formal analysis, investigation. **Mohsen Kheiri**: data curation, writing review, and editing. **Hadis Tolomehr**: data curation, writing review, and editing.

## Ethics Statement

The authors have nothing to report.

## Conflicts of Interest

The authors declare no conflicts of interest.

## Peer Review

The peer review history for this article is available at https://doi.org/10.1002/brb3.71065.

## Data Availability

The datasets used and/or analyzed during the current study are available from the corresponding author on reasonable request.
